# N-Acetylcysteine, an ROS Inhibitor, Alleviates the Pathophysiology of Hyperthyroidism-Induced Cardiomyopathy via the ROS/Ca^2+^ Pathway

**DOI:** 10.3390/biom12091195

**Published:** 2022-08-29

**Authors:** Mengni Bao, Xiumeng Hua, Han Mo, Zhe Sun, Bo Xu, Xiao Chen, Mengda Xu, Xinjie Xu, Jiangping Song

**Affiliations:** 1 Shenzhen Key Laboratory of Cardiovascular Disease, Fuwai Hospital Chinese Academy of Medical Sciences, Shenzhen 518057, China; 2 State Key Laboratory of Cardiovascular Disease, Fuwai Hospital, National Center for Cardiovascular Diseases, Chinese Academy of Medical Sciences, Peking Union Medical College, 167A Beilishi Road, Xi Cheng District, Beijing 100037, China; 3 Department of Thyroid Surgery, Guangzhou First People’s Hospital, School of Medicine, South China University of Technology, Guangzhou 518057, China

**Keywords:** hyperthyroidism-induced cardiomyopathy, NAC, ROS/Ca^2+^, fibrosis, apoptosis, hypertrophy

## Abstract

Hyperthyroidism is common and can induce cardiomyopathy, but there is no effective therapeutic strategy. The purpose of this study was to investigate the molecular mechanism of hyperthyroidism-induced cardiomyopathy (HTC) and the effect of N-acetylcysteine (NAC), an ROS inhibitor, on the pathophysiology of HTC in vivo and in vitro. Compared with those in the control groups in vivo and in vitro, TT3 and TT4 were significantly increased, the structure of myocardial cells was enlarged and disordered, and interstitial fibrosis and the apoptosis of myocardial cells were markedly increased in the L-Thy group. The ROS and inflammatory response were increased in the hyperthyroidism group. In the NAC group, the contents of TT3 and TT4 were decreased, the myocardial cell structure was slightly disturbed, fibrosis and apoptosis were significantly reduced, and the ROS level and inflammatory response were significantly reduced. Interestingly, L-Thy decreased the viability of fibroblasts and H9c2 cells, suggesting that L-Thy-induced fibrosis was not caused by the proliferation of fibroblasts. The molecular mechanism of HTC could be explained by the fact that L-Thy could cause cardiac hypertrophy, inflammation, and fibrosis by regulating the Ca^2+^/calpain/Rcan1-dependent signalling pathway, the Ca^2+^/Rcan1/NF-κB/p65-dependent signalling pathway, and the Ca^2+^/ROS/Bcl-2/caspase-3-dependent signalling pathway. In conclusion, NAC can alleviate the pathophysiology of hyperthyroidism-induced cardiomyopathy, probably by regulating the ROS/Ca^2+^-dependent pathway.

## 1. Introduction

Hyperthyroidism refers to the persistently high-functioning state of the thyroid gland, which synthesises and releases too much thyroxine, thus resulting in clinical symptoms such as thyrotoxicosis [1]. It has been reported that thyroxine can exert several effects on the cardiovascular system and influence arrhythmias, cardiac hypertrophy, and congestive heart failure [1,2]. Cardiomyopathy triggered by hyperthyroidism is called hyperthyroidism-induced cardiomyopathy (HTC), and cardiac hypertrophy is the most documented cardiomyopathy following hyperthyroidism in experimental animals and clinical practice [3,4]. Thyroid hormone-induced cardiac hypertrophy is described as a relative ventricular hypertrophy encompassing the whole heart and is linked with contractile abnormalities in both the right and left ventricles.

Regarding the mechanisms underlying the intracellular cardiac effects of thyroxine, the effects of thyroxine at the cardiac intracellular level are divided into genomic and nongenomic pathways [5]. In the genomic pathway, thyroxine regulates the expression of target genes by binding to nuclear receptors in cardiomyocytes. In contrast, the nongenomic pathway includes effects on ion channels of cardiomyocytes and effects of thyroxine on the peripheral circulation, which regulates hemodynamics and the cardiac ejection fraction [6,7]. In the nongenomic pathway, oxidative stress occurs in an experimental hyperthyroidism model, which indicates that reactive oxygen species (ROS) are key players in the cardiomyopathy frequently reported in this endocrine disorder [3]. It has been reported that thyroxine has effects on the cardiomyocytes of several membrane ion channels, such as Na^+^, K^+^, and Ca^2+^ channels [1]. The relationship between ROS and Ca^2+^ in HTC pathogenesis is unknown. In other cases, Ca^2+^ and ROS are versatile signalling molecules, coordinating physiological and pathophysiological processes, such as ageing and age-related diseases [8], heart failure [9], and hepatotoxicity [10].

N-acetyl-l-cysteine (NAC) has long been used therapeutically for the treatment of acetaminophen (paracetamol) overdose, acting as a precursor for the substrate (l-cysteine) in the synthesis of the hepatic glutathione (GSH) depleted through drug conjugation. More recently, however, many clinical studies have reported on the use of NAC as an antioxidant, most notably in the protection against ROS-dependent apoptotic processes [11,12]. In our study, we speculated that NAC could alleviate the pathophysiology of HTC via the ROS/Ca^2+^ pathway. To verify this hypothesis, we built the experimental animal and cell models to conduct experiments and try to explain the molecular mechanism of HTC.

## 2. Results

### 2.1. High L-Thy Induced the Occurrence of Cardiac Structural and Functional Changes in Mice

**Figure 1:**
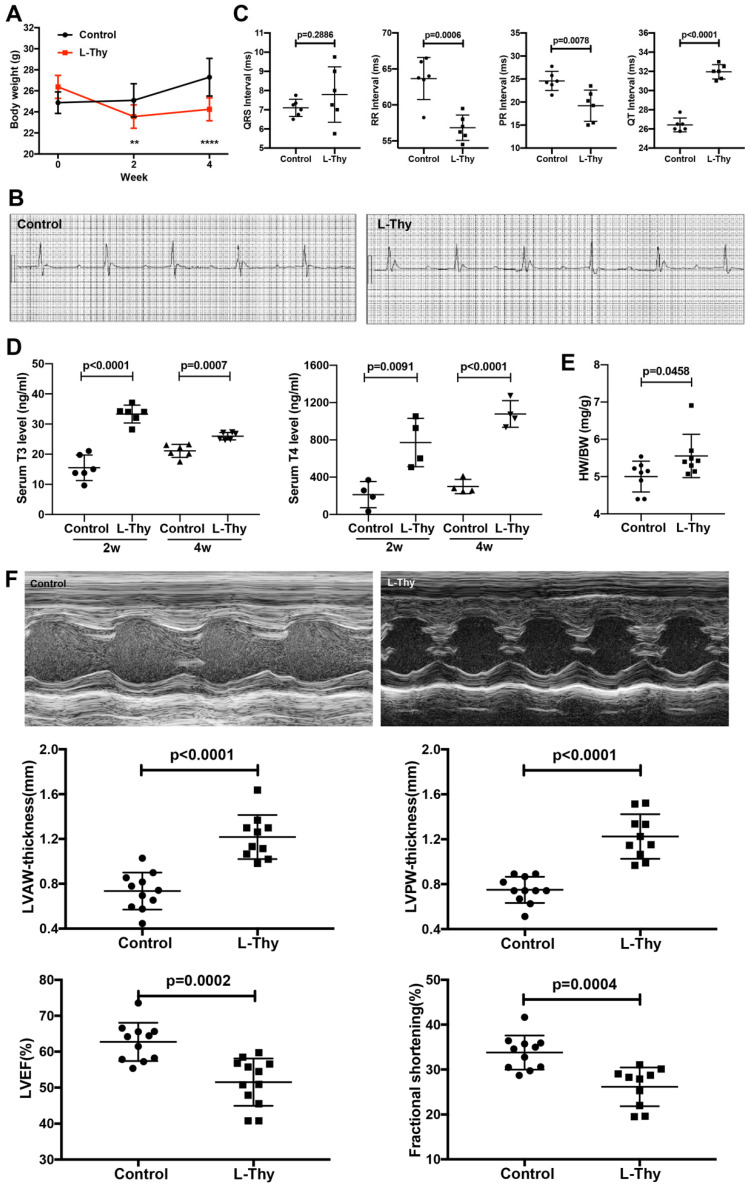
L-Thyroxine induces cardiomyopathy in mice. (**A**) Average weight in all experimental groups of C57BL/6 mice. (**B**) Representative images of electrocardiograms. (**C**) Quantification of QRS, PR, RR, and QT interval times in all experimental groups of mice. (**D**) Quantification of the content of T3 and T4 in serum. (**E**) Quantification of the heart weight to body weight (HW/BW) for all experimental groups. (**F**) Representative images of M-mode echocardiography and quantifications of end-diastolic left ventricular (LV) anterior and posterior wall thickness, ejection fraction, and fractional shortening (LVAWd, LVPWd, LVEF, and LVFS). Data are presented as the mean ± SD, ** *p* < 0.01, **** *p* < 0.0001.

First, mice were treated with L-thyroxine (L-Thy) by intraperitoneal injection daily for 4 weeks (Material and Methods) [13,14]. From the second week, L-Thy-induced mice were lighter than controls (23.55 ± 0.29 vs. 25.04 ± 0.48 g, *p* = 0.0076), and the difference in weight of both groups increased at the 4th week (24.25 ± 0.28 vs. 27.29 ± 0.54 g, *p* < 0.0001, Figure 1A). Second, after 4 weeks of induction with L-Thy, the RR and PR interval times were both decreased in the L-Thy-treated mouse group, while the QT interval time was increased in the L-Thy-treated mouse group (Figure 1B,C). The serum levels of TT3 and TT4 indicated that the levels of TT3 and TT4 in the L-Thy group were significantly higher than those in the control group at the 2nd and 4th weeks (Figure 1D). Meanwhile, the heart weight was significantly higher in the L-Thy group than in the control group (Figure 1E). Additionally, L-Thy-treated mice showed a significant increase in the left ventricular hypertrophy parameters, and LVAW and LVPW coincided with a significant reduction in the LV cardiac function markers, EF, and FS as compared to controls (Figure 1F).

**Figure 2:**
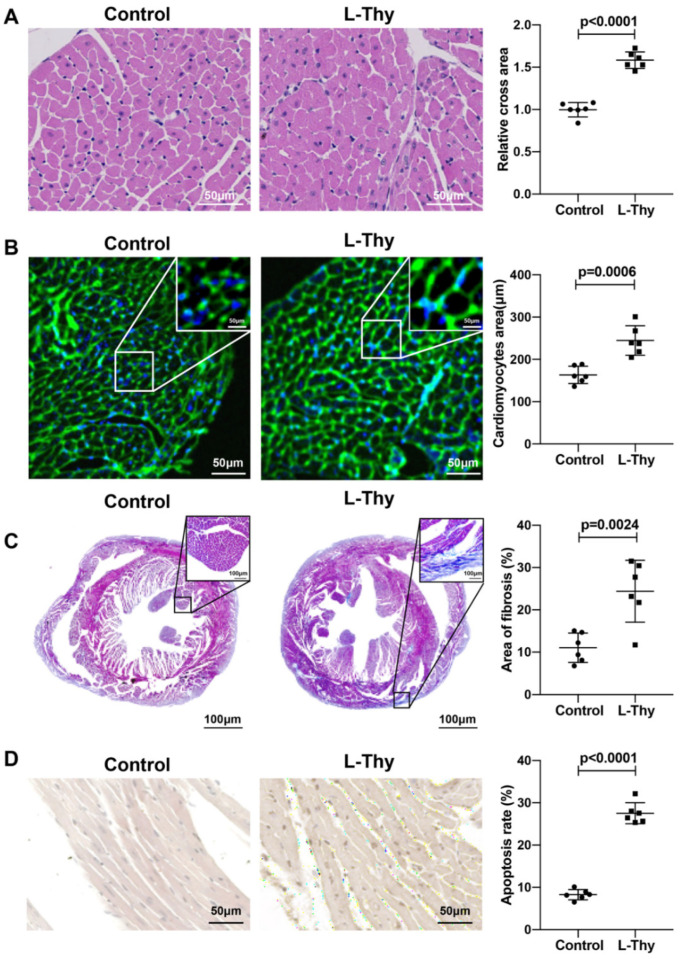
Pathological performance of hyperthyroidism-induced cardiomyopathy. (**A**–**D**) Representative images and quantification of HE-stained, WGA-stained, Masson-stained, and TUNEL-stained mice hearts.

At the same time, HE and WGA staining revealed that the L-Thy group had myocardial hypertrophy (Figure 2A,B). Moreover, Masson staining revealed that the L-Thy group had obvious fibrosis (Figure 2C), and TUNEL staining revealed that the L-Thy group had a higher cardiomyocyte apoptosis percentage (Figure 2D). Together, L-Thy could induce cardiomyopathy that was characterised by cardiac structural and functional changes, to meet mechanistic research needs.

### 2.2. Increased ROS and Inflammatory Gene Expression Was Higher in the Hyperthyroidism-Induced Cardiomyopathy Group

**Figure 3:**
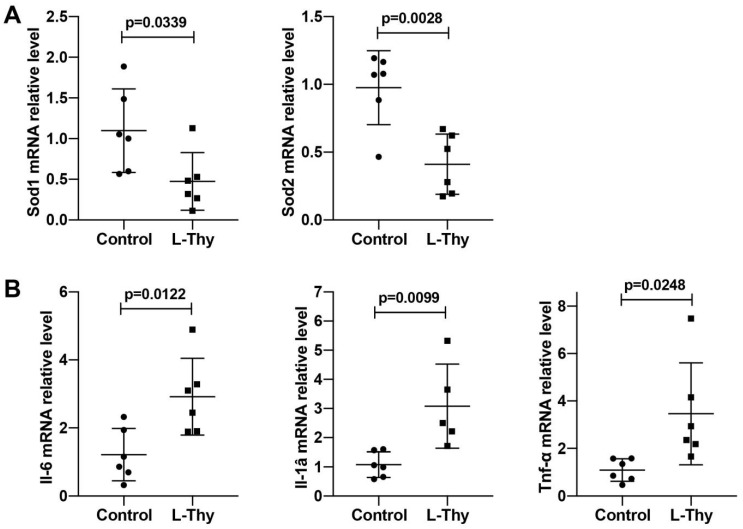
The expression of Sod and inflammatory genes in hyperthyroidism-induced cardiomyopathy. (**A**,**B**) qRT-PCR detection of Sod1, Sod2, Il-6, Il-1, and Tnf-α mRNA levels in all experimental groups of mice.

It was reported that L-Thy increases the oxidative stress response, leading to a weakened antioxidative capacity of the myocardial cells and accelerated cell damage [3]. In this study, quantitative real time-polymerase chain reaction (qRT-PCR) and Western blot (WB) showed that Sod1 and Sod2 mRNA expression was lower in the L-Thy group, suggesting that ROS expression was higher in the L-Thy group (Figure 3A and Figure S1). In addition, it has been reported that an inflammatory response occurs in hyperthyroidism-induced cardiomyopathy. In our study, we detected the expression levels of Tnf-α, Il-1β, and Il-6 in myocardial tissues, which are key indicators of the proinflammatory response. The results showed that compared with those in the control group, the mRNA expression levels of Tnf-α, Il-1β, and Il-6 in the hyperthyroidism group were significantly increased compared with those in the controls (Figure 3B and Figure S1). This suggested that L-Thy would induce the cardiac inflammatory response and oxygen free radical production.

### 2.3. Hyperthyroidism-Induced Cardiomyopathy Cell Model for Mechanistic Investigation

**Figure 4:**
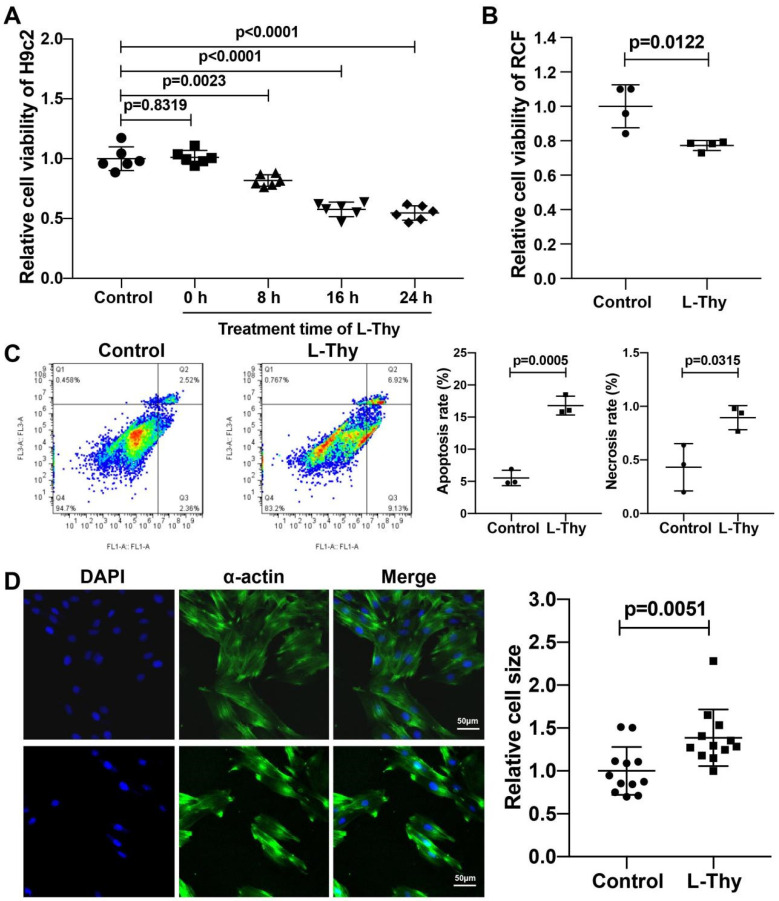
Hyperthyroidism-induced cardiomyopathy cell model. (**A**) Alterations in the viability of H9c2 cardiac cells treated with L-Thy for different times determined by Cell Counting Kit-8 assay. (**B**) Alterations in the viability of RCFs treated with L-Thy. (**C**) Typical apoptotic and necrotic pictures of different experimental groups (Q1: necrotic cells; Q2: medium- or late-stage apoptotic cells; Q3: early-stage apoptotic cells; Q4: normal cells) and quantification of the apoptosis rate and necrosis rate in different groups. (**D**) Representative image and surface area quantification of α-actin-stained H9c2 cardiac cells treated with L-Thy for 24 h. Scale bar, 50 μm. Dots represent the fields of vision. *n* > 150 cells.

To further elucidate the mechanism, we built a cell model. The H9c2 cell line was also treated with 2 μM L-Thy [13], and we found that L-Thy decreased the viability of H9c2 cells, as detected by the CCK-8 assay (Figure 4A). To explain why fibrosis increases in L-Thy-induced cardiomyopathy (Figure 2C), we also treated the rat cardiac fibroblasts (RCF) with L-Thy. The results showed that L-Thy also decreased the cell viability of fibroblasts, indicating that fibrosis was not caused by the proliferation of fibroblasts treated with L-Thy in L-Thy-induced cardiomyopathy (Figure 4B). To verify the apoptosis and necrosis of H9c2 cells, flow cytometry was used to detect that the necrosis and apoptosis percentages of the H9c2 cell line were higher in the L-Thy group than in the control group (Figure 4C). In addition, the immunofluorescence of α-actin results suggested that the cell size of L-Thy-treated H9c2 cells was larger than that of control cells (Figure 4D).

### 2.4. ROS/Ca^2+^ Pathway and Inflammatory Gene Expression in a Hyperthyroidism-Induced Cardiomyopathy Cell Model, Consistent with the Performance of the Animal Model

**Figure 5:**
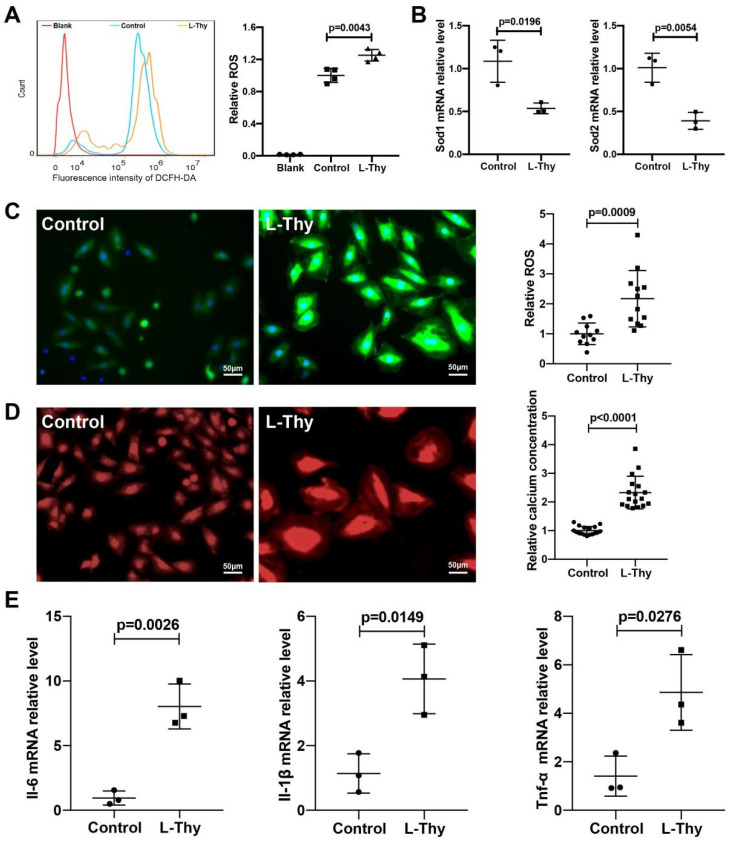
ROS/Ca^2+^ pathway and inflammatory gene expression in the hyperthyroidism-induced cardiomyopathy cell model. (**A**,**C**) Representative images and quantification of ROS levels in H9c2 cardiac cells determined by FCM and confocal microscopy. Scale bar, 50 μm. (**B**,**E**) The mRNA levels of Sod1, Sod2, Il-6, Il-1, and Tnf-α measured by qRT-PCR. (**D**), Representative images and intracellular Ca^2+^ concentration quantification of Rhod-4 stained H9c2 cells. Scale bar, 50 μm.

Furthermore, the ROS level was detected by flow cytometry, which showed that the ROS level in the L-Thy group was higher than that in the control (Figure 5A), and this was consistent with the animal model (Figure 3A). Similarly, Sod1 and Sod2 mRNA expression was lower in the L-Thy group (Figure 5B). Due to the relationship of ROS and Ca^2+^ in cardiovascular disease, we detected Ca^2+^ by immunofluorescence of Rhod-4, which suggested that Ca^2+^ increased in the L-Thy group (Figure 5C). At same time, the cell model also indicated that L-Thy could induce a cardiac inflammatory response in which Il-1β, Tnf-α, and Il-6 mRNA expressions were increased in the L-Thy-treated H9c2 cell line (Figure 5D). Together, cell lines and animal models suggested that the ROS/Ca^2+^ pathway contributed to L-Thy-induced cardiomyopathy.

### 2.5. NAC Could Relieve L-Thy-Induced Myocardial Hypertrophy and the Cardiac ROS/Ca^2+^ Pathway

**Figure 6:**
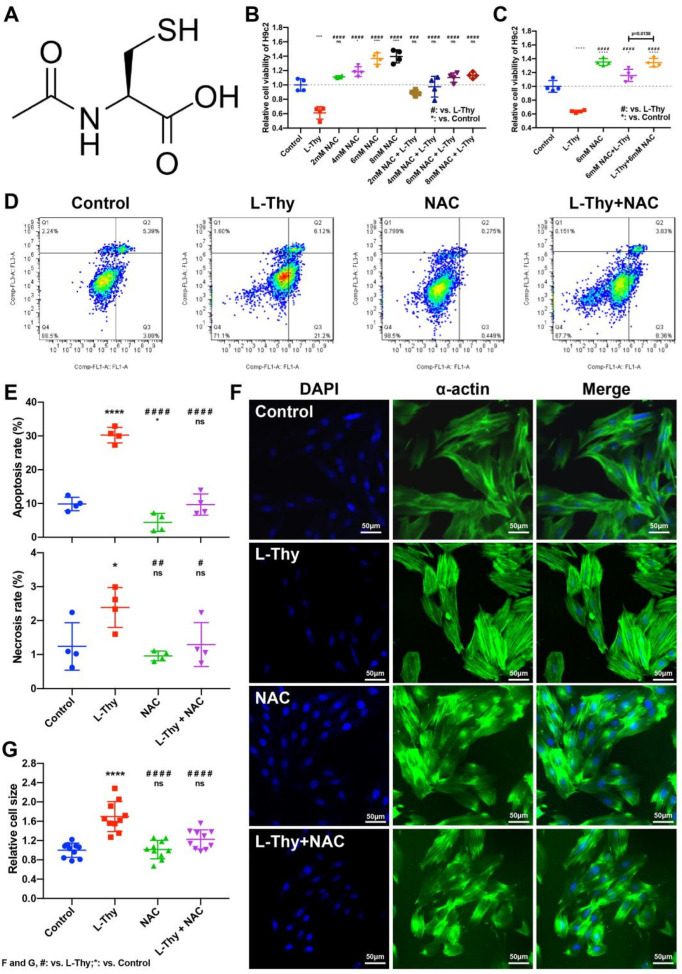
NAC attenuated the phenotype of the hyperthyroidism-induced cardiomyopathy cell model. The H9c2 cells in the logarithmic growth phase were plated. The control group was cultured in normal medium, and the L-Thy group was treated with 2 μM L-Thy for 24 h. (**A**) The structural formula of NAC. (**B**) Alterations in the viability of H9c2 cardiac cells treated with NAC at different concentrations, as determined by Cell Counting Kit-8 assay. The 2/4/6/8 mM NAC group was cultured in normal medium and then treated with different concentrations of NAC for 6 h. In the L-Thy + 2/4/6/8 mM NAC group, H9c2 cells were previously incubated with 2 μM L-Thy for 24 h and then maintained in medium containing different concentrations of NAC for 6 h. (**C**) Alterations in the viability of H9c2 cells treated with NAC before or after cell modelling. The 6 mM NAC group was treated with 6 mM NAC for 6 h. In the L-Thy + 6 mM NAC group, H9c2 cells were previously incubated with 2 μM L-Thy for 24 h and then maintained in 6 mM NAC-containing medium for 6 h. In the 6 mM NAC + L-Thy group, H9c2 cells were previously incubated with 6 mM NAC-containing medium for 6 h and then maintained in 2 μM L-Thy-containing medium for 24 h. (**D**–**G**) The control group was cultured in normal medium, and the L-Thy group was treated with 2 μM L-Thy for 24 h. In addition, the NAC group was treated with 6 mM NAC for 6 h. In the L-Thy + NAC group, H9c2 cells were previously incubated with 2 μM L-Thy for 24 h and then maintained in 6 mM NAC-containing medium for 6 h. (**D**,**E**) Typical apoptotic and necrosis pictures and quantification of the apoptosis rate and necrosis rate in different groups. (**F**,**G**) Representative image and surface area quantification of α-actin-stained H9c2 cardiac cells. Scale bar, 50 μm. */# *p* < 0.05, ## *p* < 0.01, ### *p* < 0.001, and ****/#### *p* < 0.0001.

To verify the role of the ROS/Ca^2+^ pathway in L-Thy-induced cardiomyopathy, we used a small molecular ROS inhibitor, NAC [15], to treat the L-Thy-treated H9c2 cell line (Figure 6A). First, we evaluated different concentrations to identify the most appropriate one, finding that there was a dose-response effect at first, but later, when the concentration of NAC increased from 6 mM to 8 mM, there was no significant change in cell viability (*p* = 0.4451) (Figure 6B). Then, we compared the time of giving treatment before disease and after disease. We found that the effect of treatment was better than prevention at a concentration of 6 mM (*p* = 0.0138) (Figure 6C). Furthermore, NAC could relieve myocardial necrosis and the apoptosis percentage (Figure 6D,E). According to the immunofluorescence of α-actin, NAC significantly relieved L-Thy-induced myocardial hypertrophy (Figure 6F,G). NAC, an ROS inhibitor, could relieve L-Thy-induced myocardial hypertrophy in the H9c2 cell line.

**Figure 7:**
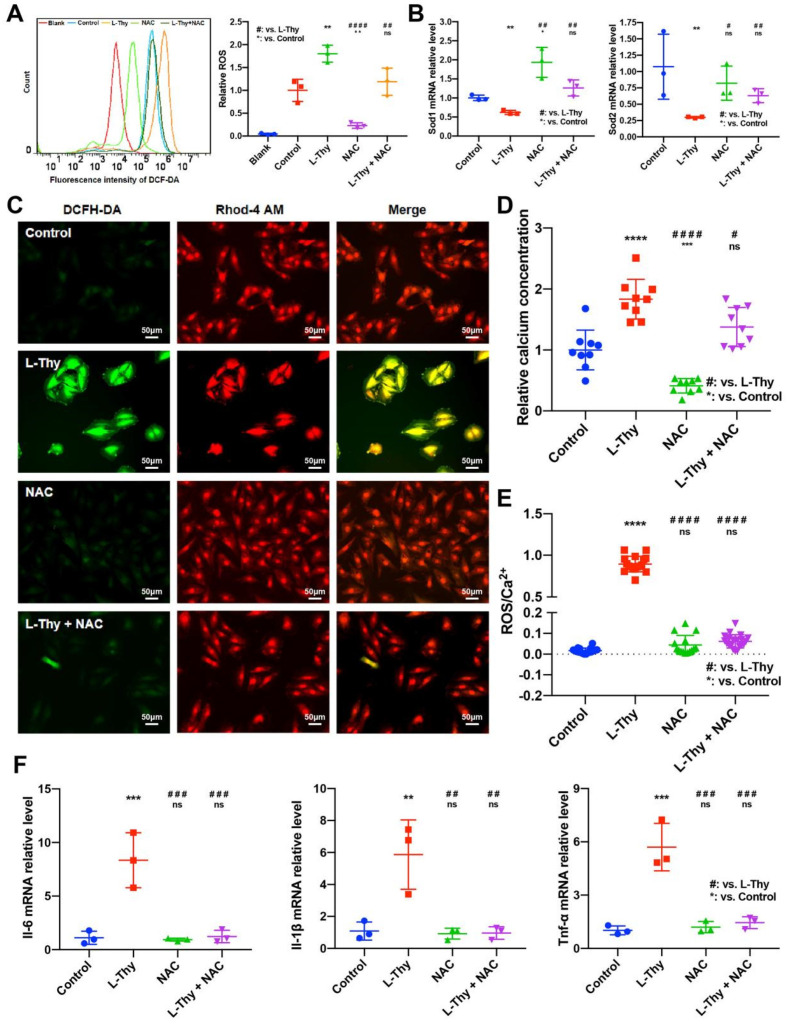
NAC attenuated the ROS/Ca^2+^ pathway in a hyperthyroidism-induced cardiomyopathy cell model. H9c2 cells in logarithmic growth phase were plated. The control group was cultured in normal medium, and the L-Thy group was treated with 2 μM L-Thy for 24 h. In addition, the NAC group was treated with 6 mM NAC for 6 h. In the L-Thy + NAC group, H9c2 cells were previously incubated with 2 μM L-Thy for 24 h and then maintained in 6 mM NAC-containing medium for 6 h. (**A**) Representative images and quantification of ROS levels in H9c2 cells determined by FCM. (**B**) The mRNA levels of Sod1and Sod2 measured by qRT-PCR. (**C**–**E**) Representative images, quantification of DCFH-DA/Rhod-4-stained H9c2 cells and ratio of Ros/Ca^2+^ in different groups. Scale bar, 50 μm. The intracellular ROS and calcium contents were expressed by the fluorescence intensity of DCFH-DA and Rhod-4, respectively. Fluorescence intensity was quantified by the ImageJ plugin 3D Object Counter [16,17]. (**F**) The mRNA levels of Il-6, Il-1, and Tnf-α were measured by qRT-PCR. */# *p* < 0.05, **/## *p* < 0.01, ***/### *p* < 0.001, and ****/#### *p* < 0.0001.

Furthermore, we detected the expression of ROS/Ca^2+^ pathway components during NAC treatment of L-Thy-induced myocardial changes. ROS levels were decreased, while Sod1 and Sod2 mRNA expression were increased in the NAC-treated L-Thy group (Figure 7A,B). The immunofluorescence results showed that the ROS/Ca^2+^ pathway could be inhibited by NAC in L-Thy-induced myocardial hypertrophy (Figure 7C–E). Moreover, the inflammatory response was also inhibited, which indicated that the L-Thy-induced cardiac inflammatory response was regulated by the ROS/Ca^2+^ pathway (Figure 7F). Together, NAC could relieve L-Thy-induced myocardial hypertrophy and inflammation via the ROS/Ca^2+^ pathway in vitro.

**Figure 8:**
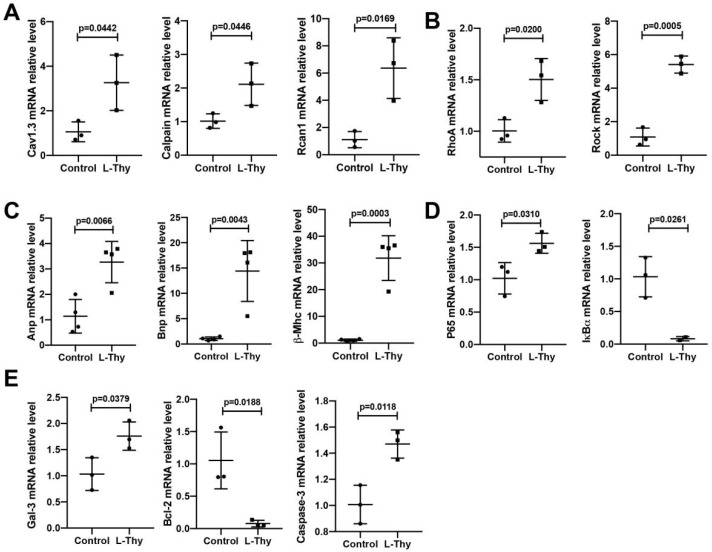
L-Thy-induced phenotypes were explained from the perspective of signalling pathways. (**A**–**E**) qRT-PCR was used to detect Cav1.3, calpain, Rcan1, RhoA, Rock, Anp, Bnp, β-Mhc, P65, IκBα, Gal-3, Bcl-2, and Caspase-3 mRNA levels.

To explain the molecular mechanism of HTC, we used qRT-PCR to verify the classical ROS/Ca^2+^ signalling pathways, on the basis of previous studies. We have shown here that the gene expression of L-type Cav1.3 Ca^2+^ channels (Cav1.3), calpain [18], regulator of calcineurin 1 (Rcan1), RhoA, Rho-kinase (Rock), atrial natriuretic peptide (Anp), brain natriuretic peptide (Bnp), β-myosin heavy chain (β-Mhc), p65, galectin-3 (Gal-3), and caspase-3 increased with decreases in the inhibitor of NF-κB α (IκBα), and the B-cell lymphoma-2 (Bcl-2) genes in H9c2 cells were treated with L-Thy (Figure 8 and Figure S2). In addition, calcium antagonists (Isradipine) could reduce L-Thy-induced myocardial damage (Figure S3). Together, ROS and calcium antagonists could relieve L-Thy-induced cardiac damage.

### 2.6. NAC Attenuated the Phenotype of Hyperthyroidism-Induced Cardiomyopathy In Vivo

**Figure 9:**
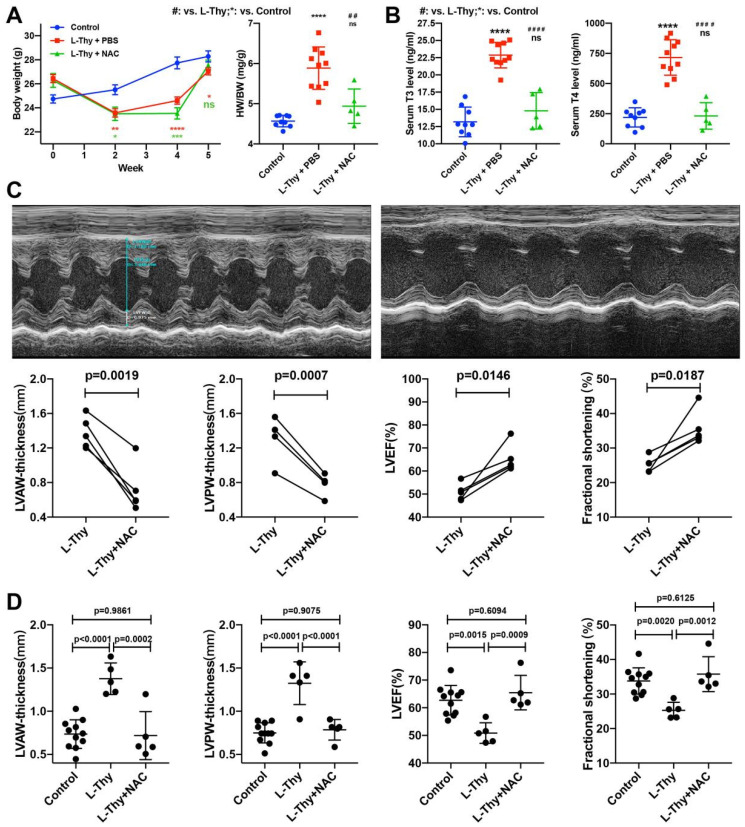
NAC could attenuate the cardiac function of hyperthyroidism-induced cardiomyopathy in vivo. (**A**) Average weight and quantification of the heart weight to body weight (HW/BW) in all experimental groups of mice. (**B**) Quantification of the T3 and T4 contents in the serum of all experimental groups of mice. (**C**,**D**) Representative images of M-mode echocardiography and quantifications of LVAWd, LVPWd, LVEF, and LVFS. */# *p* < 0.05, **/## *p* < 0.01, ***/### *p* < 0.001, and ****/#### *p* < 0.0001.

**Figure 10:**
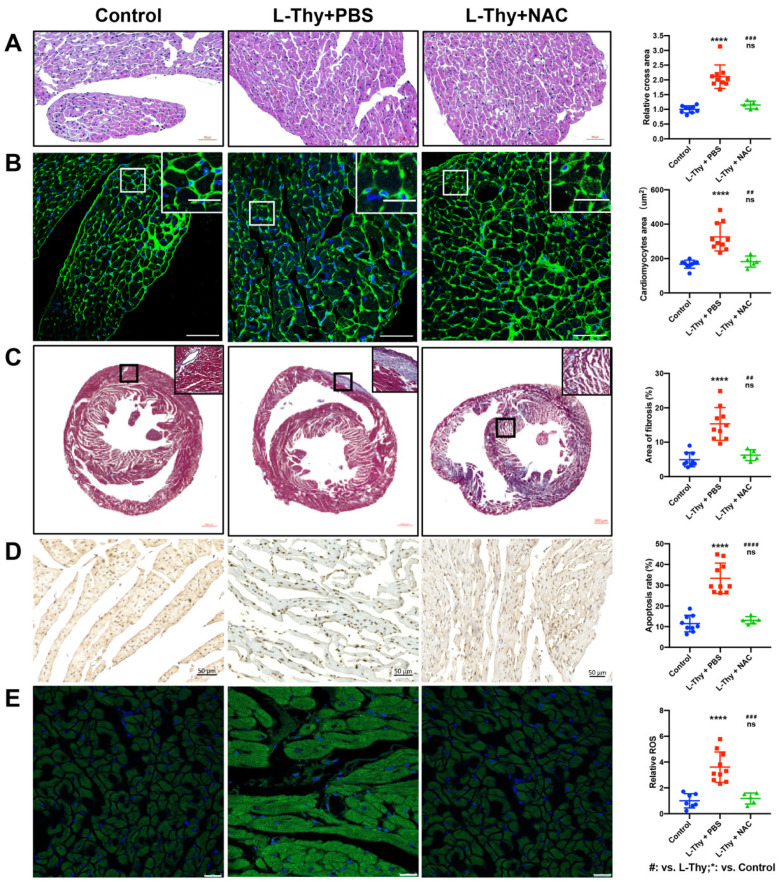
NAC attenuated the pathological phenotype of hyperthyroidism-induced cardiomyopathy in vivo. (**A**–**E**) Representative images and quantification of HE-stained, WGA-stained, Masson-stained, TUNEL-stained, and DCFH-DA-stained mouse hearts. ## *p* < 0.01, ### *p* < 0.001, and ****/#### *p* < 0.0001.

According to in vitro experiments, NAC relieved L-Thy-induced myocardial pathophysiological changes via the ROS/Ca^2+^ pathway. Furthermore, NAC was applied to treat L-Thy-induced cardiomyopathy to investigate whether inhibition of the ROS/Ca^2+^ pathway could attenuate the phenotype of hyperthyroidism-induced cardiomyopathy. After 1 week of intraperitoneal injection of NAC in the L-Thy-induced cardiomyopathy model, we found that NAC could attenuate the loss of body weight, ratio of HW to BW, and content of TT3 and TT4 (Figure 9A,B). In addition, after NAC treatment, the myocardial hypertrophy of diseased mice decreased, and cardiac function improved compared to before treatment (Figure 9C). Moreover, NAC could attenuate myocardial hypertrophy, in which LVAW and LVPW thickness were decreased in the L-Thy + NAC group compared with the L-Thy group, and cardiac function, in which LVEF and FS increased in the L-Thy + NAC group compared with that of the L-Thy group (Figure 9D). In addition, morphological assessment of the cardiac tissue of L-Thy + NAC-treated mice showed normal myocardial size, less fibrosis, a lower apoptosis percentage, and lower ROS levels than L-Thy-induced cardiomyopathy mice (Figure 10). Together, inhibition of the ROS/Ca^2+^ pathway was the potential target of hyperthyroidism-induced cardiomyopathy.

## 3. Discussion

The results reported in our study suggest that hyperthyroidism induced by chronic administration of L-Thy in mice and the H9c2 cell line was an independent factor that affected cardiac structure and function and then led to adverse remodelling, fibrosis, and inflammation. Meanwhile, our data suggested that these effects may be mediated by the ROS/Ca^2+^ pathway. Interestingly, scavenging ROS with NAC protected against L-Thy-induced impairment in cardiac function and associated cardiac damage, by alleviating all the pro-oxidant, inflammatory, and fibrotic events. We were surprised to find that in animal experiments, NAC could not only improve the cardiac function and pathology of HTC by reducing ROS but also reduce the contents of TT3 and TT4 in serum. There are two pathways that NAC plays roles in the pathogenesis of HTC: one is that NAC reduces ROS and, thus, improves the cardiac function and pathology, and the other is that NAC reduces the content of TT3 and TT4 via the decreased level of ROS and, thus, improves the cardiac function and pathology of HTC. Then, in the cell model, we found that NAC could ameliorate myocardial injury by reducing ROS but not reduce TT3 and TT4 levels. ROS have been documented to promote hyperthyroidism [19], so we can speculate that NAC reduces the levels of TT3 and TT4 via the decreased level of ROS.

**Figure 11:**
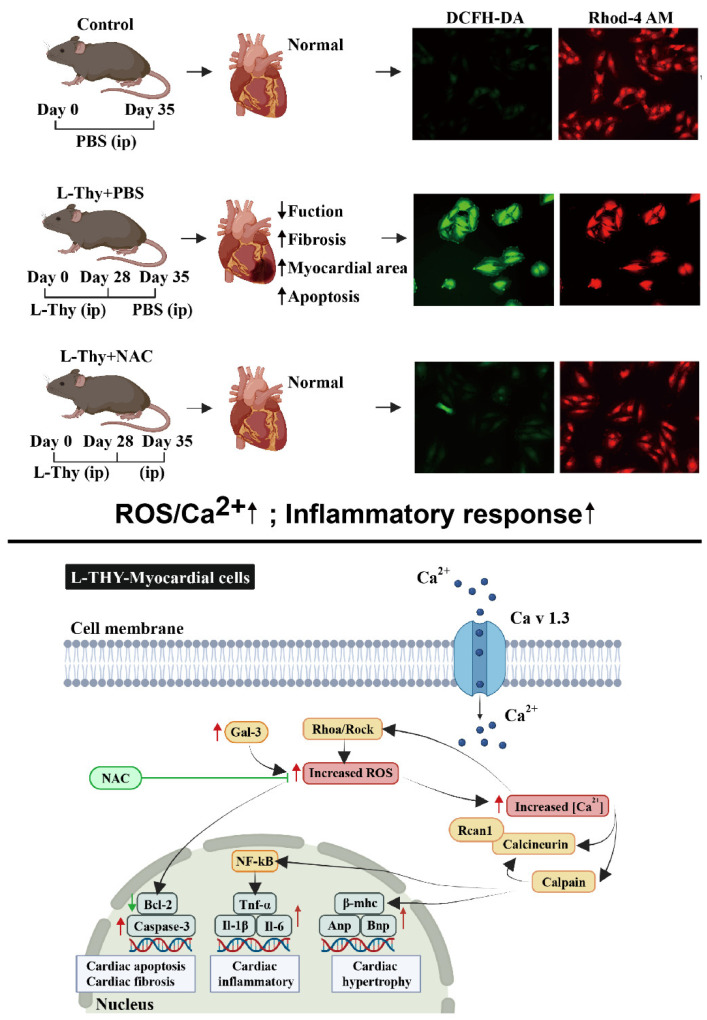
Graphical abstract for summarizing the contents of the article. Under the stimulation of hyperthyroidism, extracellular calcium ions flow through L-type calcium channel Cav1.3, which promotes the activation of calpain and calcineurin mediated by calcium ions, thereby promoting the transcriptome transition toward cardiac hypertrophy and myocardial inflammation. At the same time, ROS level increases in cardiomyocytes and activates the expression of Bcl-2/Caspase signalling pathway, thereby promoting cardiomyocyte injury. NAC can inhibit ROS level then decrease the apoptosis of cardiomyocytes to ensure the safety of the heart.

Administration of L-Thy by intraperitoneal injection remained the best strategy to induce hyperthyroidism to induce cardiomyopathy in rodents [13,20]. In our study, we evaluated our animal and cell models from different aspects, such as serum thyroxine levels, body weight, heart mass, cardiac structure and function, and histopathologic alterations. Combining these results, the animal and cell model simulated the process of heart disease caused by hyperthyroidism, which could be applied to investigate the molecular mechanism. As shown in Figure 11, an increase in Cav1.3 induces an increase in the cytosolic Ca^2+^ concentration of H9c2 cells, thereby increasing the activation of Ca^2+^-dependent signalling, Rcan1, calcineurin, and calpain, which subsequently upregulates the gene expression of hypertrophy markers and upregulates the gene expression of inflammatory cytokines by activating the NF-κB/p65-dependent signalling pathway [21,22,23,24,25,26,27,28], causing cardiac hypertrophy and inflammation. Meanwhile, the elevation of Gal-3, RhoA, and Rock further increased ROS [27,28,29,30,31,32], subsequently regulating the Bcl-2/caspase-3-dependent apoptotic signalling pathway [33,34], thereby leading to cell rupture and fibrosis.

In our study, ROS played critical roles in HTC, consistent with a previous report that showed that ROS were the central mechanism of hyperthyroidism-induced cardiomyopathy that was characteristic of myocardial hypertrophy, apoptosis, inflammation, and arrhythmia [20]. ROS and Ca^2+^ are versatile signalling molecules that coordinate physiological and pathophysiological processes [8]. In our study, we found that the ROS/Ca^2+^ pathway contributed to the pathogenesis of HTC. This result suggested that the Ca^2+^ antagonist nifedipine could be applied for treating HTC patients in situations where the application value of Ca^2+^ antagonists in HTC is controversial [35,36]. In the future, more clinical studies should be conducted to investigate how to use Ca^2+^ antagonists to treat HTC. Besides, the detailed mechanism of L-Thy-ROS/Ca^2+^ signalling pathway in HTC was not elucidated, which is the limitation of this study.

Untreated hyperthyroidism is associated with increased cardiovascular morbidity and mortality [37]. Under this circumstance, the clinical question arises of whether to administer prophylactic drugs to treat hyperthyroidism-associated cardiac injury. We found that prophylactic administration was not beneficial for hyperthyroidism-induced cardiomyopathy (Figure 6C). Therefore, prophylactic drugs are not recommended to protect the heart in hyperthyroidism patients.

## 4. Conclusions

Our data showed that cardiac structural and functional changes were related to the ROS/Ca^2+^ pathway. In addition, these data showed that thyroid hormones would induce ROS overproduction and then induce Ca^2+^ release to cause myocardial hypertrophy, apoptosis, fibrosis, and inflammation, while inhibition of ROS could regulate the ROS/Ca^2+^ signalling pathways to alleviate HTC.

## 5. Methods

### 5.1. Cell Culture

H9c2(2-1) (ATCC CRL-1446), and RCF (HTX2988) cells were cultured in Dulbecco’s Modified Eagle Medium (DMEM, Gibco) supplemented with 10% foetal bovine serum (FBS, Gibco) in 5% CO_2_ at 37 °C. When the cell density reached approximately 80%, the cells were digested with 0.25% trypsin every two to three days.

### 5.2. Hyperthyroid Cardiomyopathy Model Induction

Eight-week-old male C57BL/6 mice (Vital River Laboratories, Beijing, China) were maintained in a specific pathogen-free facility and provided a normal diet and drinking water. After 1 week of adaptive feeding, the mice were intraperitoneally injected with 100 μg/kg L-Thy (Merck, Darmstadt, Germany) compatible in solution with sodium carboxymethylcellulose (C4888, Sigma, Shanghai, China) every day. The mice were examined with echocardiography and euthanised on Day 28. Meanwhile, the weight and heart rate of the mice were measured. This investigation was approved by the Animal Ethics Committee of Fuwai Hospital (FW-2022-0015).

When the cell density reached approximately 60%, H9c2 cells were incubated with 2 μM L-Thy at 37 °C for 24 h.

### 5.3. Experimental Grouping

These experimental animals were randomly divided into three groups: the control group, L-Thy + PBS group, and L-Thy + NAC group. The control group was intraperitoneally injected with PBS for 35 days. In the L-Thy + PBS group and L-Thy + NAC group, mice were injected intraperitoneally with L-Thy for 28 days. In the L-Thy + NAC group, after the hyperthyroid cardiomyopathy model was established, 200 mg/kg NAC (HY-B0215, MCE, Shanghai, China) was continuously injected for 7 days. In addition, the L-Thy + PBS group was given the same amount of PBS daily.

H9c2 cells in the logarithmic growth phase were plated. The control group was cultured in normal medium, and the L-Thy group was treated with 2 μM L-Thy for 24 h. In addition, the NAC group was treated with 6 mM NAC for 6 h. In the L-Thy + NAC group, H9c2 cells were previously incubated with L-Thy and then maintained in NAC-containing medium for 6 h.

### 5.4. Haematoxylin-Eosin (HE) and Masson Staining

After cardiac perfusion, the hearts were removed quickly from the mice, wiped softly with clean absorbent paper, and cut in half horizontally with a knife. Half of the heart tissues were taken using paraformaldehyde, and the remaining tissues were stored at −80 °C.

The fixed mouse heart tissues were cut into 4 μm thick sections, stained in accordance with the instructions of the kit, sealed with a sealing liquid, and finally observed with a pathologic scanner (Zeiss, Oberkochen, Germany).

### 5.5. TUNEL Assay

Paraffin-embedded heart sections were deparaffinised, rehydrated, permeated, and antigen-retrieved, in accordance with the instructions of the TUNEL assay kit (11684795910, Roche, Shanghai, China). Sections were detected by the pathologic scanner.

### 5.6. Cell Counting Kit-8 (CCK-8) Assay

Cells inoculated in a 96-well plate were incubated with L-Thy at different time points and then not treated or treated with NAC at different concentrations (0 mM, 2 mM, 4 mM, 6 mM, 8 mM) for the next day. Otherwise, the cells were not pretreated or pretreated with NAC, and L-Thy was added. Then, CCK-8 reagent (CCK8-500, Hanbio, Shanghai, China) was added to the cells in the dark. After incubation at 37 °C for 3 h, the plate was detected at 450 nm by a microplate reader.

### 5.7. WGA, FITC-Phalloidin, DCFH-DA, and Rhod-4 Staining

To visually display the myocardial cell area, α-actin, ROS, cytosolic Ca^2+^, 20 μg/mL WGA (L4895, Sigma, Shanghai, China), 20 μg/mL FITC-phalloidin (ab235137, Abcam, Shanghai, China), 5 μM 2,7-dichlorodihydrofluorescein diacetate (D6883, DCFH-DA, Sigma, Shanghai, China), and 2.5 mM Rhod-4 (T0404, Warbio, Nanjing, China) were used to stain for 30 min in accordance with the instructions and then washed with PBS.

### 5.8. Flow Cytometry

To detect ROS levels, H9c2 cells were prepared as a single-cell suspension, and DCFH-DA was added, followed by incubation at 37 °C for 30 min in the dark. Following centrifugation, the supernatant was discarded, 10% FBS was added, and the cells were incubated for 20 min. After centrifugation, the supernatant was replaced with an appropriate amount of cold PBS.

To analyse apoptosis, H9c2 cells were trypsinised, centrifuged, collected, and washed with cold PBS to concentrate to 1 × 10^5^ cells/mL. A 100-ul sample solution was mixed with 5 µL of annexin V-FITC (V13242, AV-FITC, Thermo Fisher Scientific, Shanghai, China) and 5 µL of propidium iodide (V13242, PI, Thermo Fisher Scientific, Shanghai, China), then incubated for 15 min at room temperature, and finally washed with cold PBS.

### 5.9. Quantitative Real Time-Polymerase Chain Reaction (qRT-PCR)

Total RNA was extracted from the heart tissues stored at −80 °C and H9c2 cells in each group by TRIzol (15596018, Thermo Fisher Scientific, Shanghai, China). After quantification using a NanoDrop, 2 µg of total RNA was reverse-transcribed into complementary deoxyribose nucleic acid cDNA. The transcribed cDNA was analysed by real-time PCR with SYBR-Green dye (4472908, Thermo Fisher Scientific, Shanghai, China). The relative expression of a target gene was calculated using the 2^−ΔΔct^ method. The primers used are shown in Table 1.

### 5.10. Western Blot (WB)

The heart tissues and H9 c2 cells in each group were added with RIPA Lysis Buffer (Beyotime) and homogenised on ice. After centrifugation, the supernatant was taken and denatured by protein-loading buffer. After being electrophoresed, the protein was transferred to the nitrocellulose membrane (IB24001, Thermo Fisher Scientific, Shanghai, China), then blocked with skim milk powder, and incubated with primary antibodies (anti-SOD1 (Bioss), anti-SOD2 (Bioss), anti-Calcipressin 1/DSCR 1 (Bioss), anti-RhoA (Bioss), anti-Calpain 2 (Bioss), anti-Caspase-3 (Bioss), anti-phospho-NFKB p65 (Ser281) (Bioss), and anti-Bcl-2 (Bioss)) overnight. Next day, the corresponding secondary antibody was used for continuous incubation, the images were collected and processed after exposure to enhanced chemiluminescence (ECL), and gapdh/β-actinwas used as an internal control. The protein expression level was analysed with ECL-plus reagent (GE Healthcare, Shanghai, China).

### 5.11. Data Analysis

The images and flow cytometry results were analysed with ImageJ software and FlowJo software, respectively. All data are presented as the mean ± SD. Differences between two groups were analysed using Student’s t-test. Comparisons between multiple groups were performed using one-way ANOVA followed by a post hoc test (least significant difference). *p* < 0.05 was considered to indicate a statistically significant difference.

## Figures and Tables

**Table 1:** Primers used in this study.

Primer	Forward/Reverse Primer (5′-3′)
H9c2-Il-6-F	CCAGCCAGTTGCCTTCTTG
H9c2-Il-6-R	CAATCAGAATTGCCATTGCAC
H9c2-Il-1β-F	ACAGTGCATCATCGCTGTTC
H9c2-Il-1β-R	GAAGAATCTATACCTGTCCT
H9c2-Tnf-α-F	CTTTTCCATCTTCTTCTTTG
H9c2-Tnf-α-R	TGCTTGTTCCTCAGCCTCTT
H9c2-Sod1-F	TGGGCTACAGGCTTGTCACT
H9c2-Sod1-R	AACCAGTTGTGGTGTCAGGA
H9c2-Sod2-F	CTCCTGAGAGTGAGATCACA
H9c2-Sod2-R	TTCTGGACAAACCTGAGCCCTAA
H9c2-β-actin-F	GAACCTTGGACTCCCACAGACAC
H9c2-β-actin-R	CCCTAAGGCCAACCGTGAAAAG
H9c2-Cav1.3-F	TTGGTACGGACGGCTCTCA-
H9c2-Cav1.3-R	CCCCACGGTTACCTCATCAT
H9c2-Calpain-F	CAAAGTGGACCCCTATGAACG
H9c2-Calpain-R	TAAGGGCGTCAGGTGTAAGGT
H9c2-Rcan1-F	CTTCAGCAACCCCCTGTC
H9c2-Rcan1-R	ACTGGGGTAGCGTCTTCT
H9c2-RhoA-F	TCGGAATGATGAGCACACAA
H9c2-RhoA-R	GCTTCACAAGATGAGGCAC
H9c2-Rock-F	GTGATGGCTATTATGGACG
H9c2-Rock-R	AGGAAGGCACAAATGAGAT
H9c2-Anp-F	GATCTGCCCTCTTGAAAAGC
H9c2-Anp-R	CCAGGAGGGTATTCACCAC
H9c2-Bnp-F	CACGATGCAGAAGCTGCTGG
H9c2-Bnp-R	ACAACCTCTGCCCGTCACA
H9c2-β-Mhc-F	GACAGGAAGAACCTACTGCG
H9c2-β-Mhc-R	CTCCAGGTCTCAGGGCTTCAC
H9c2-p65-F	AGAGCAACGATTCCACCAA
H9c2-p65-R	GCAGTCTTTTCCCACCAGC
H9c2-IκBα-F	CACTCCATCCTGAAGGCTACCAA
H9c2-IκBα-R	AAGGGCAGTCCGGCCATTA
H9c2-Gal-3-F	AGCCCAACGCAAACAGTATC
H9c2-Gal-3-R	GGCTTCAACCAGGACCTGTA
H9c2-Bcl-2-F	GCCTTCTTTGAGTTCGGTG
H9c2-Bcl-2-R	GAAATCAAACAGAGGTCGC
H9c2-Caspase-3-F	GGTATTGAGACAGACAGTGG
H9c2-Caspase-3-R	CATGGGATCTGTTTCTTTGC
Mice-Il-6-F	TACGTACATGGCTGGGGTGT
Mice-Il-6-R	TAGTCCTTCCTACCCCAATTTCC
Mice-Il-1β-F	TTGGTCCTTAGCCACTCCTTC
Mice-Il-1β-R	AGGCAAACCGTGAAAAGATG
Mice-Tnf-α-F	ATCTTTTGGGGTCCGTCAACT
Mice-Tnf-α-R	CCTCACACTCAGATCATCTTCT
Mice-Sod1-F	GCTACGACGTGGGCTACAG
Mice-Sod1-R	GAGACCTGGGCAATGTGACT
Mice-Sod2-F	AACTCAGGTCGCTCTTCAGC
Mice-Sod2-R	TTGTTTCTCATGGACCACCA
Mice-β-actin-F	GCTTGATAGCCTCCAGCAAC
Mice-β-actin-R	AGGCAAACCGTGAAAAGATG

## Data Availability

Not applicable.

## Supplementary Materials

The following supporting information can be downloaded at: https://www.mdpi.com/article/10.3390/biom12091195/s1. Figure S1: The expression of SOD and TNF-α protein in hyperthyroidism-induced cardiomyopathy; Figure S2: The expression of proteins about signalling pathways in L-Thy induced phenotypes; Figure S3: Calcium antagonists (Isradipine) reduce heart muscle damage induced by high thyroid hormones.
