# Supplementary material for: N-Acetylcysteine, an ROS Inhibitor, Alleviates the Pathophysiology of Hyperthyroidism-Induced Cardiomyopathy via the ROS/Ca2+ Pathway
Source: Biomolecules. 2022 Aug 29;12(9):1195. doi: 10.3390/biom12091195 (PMC9496499; doi:10.3390/biom12091195)
Supplement: Supplementary file 1 [file biomolecules-12-01195-s001.zip › biomolecules-1864651-supplementary.pdf]

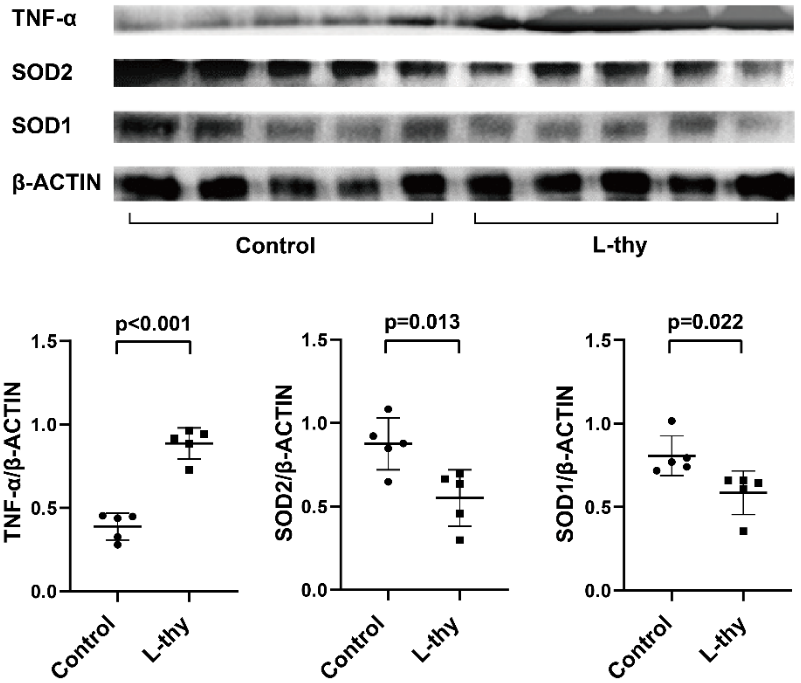

Figure S1. The expression of SOD and TNF-α protein in hyperthyroidism-induced cardiomyopathy. Protein detection of SOD1, SOD2 and TNF-α protein levels in all experimental groups of mice.

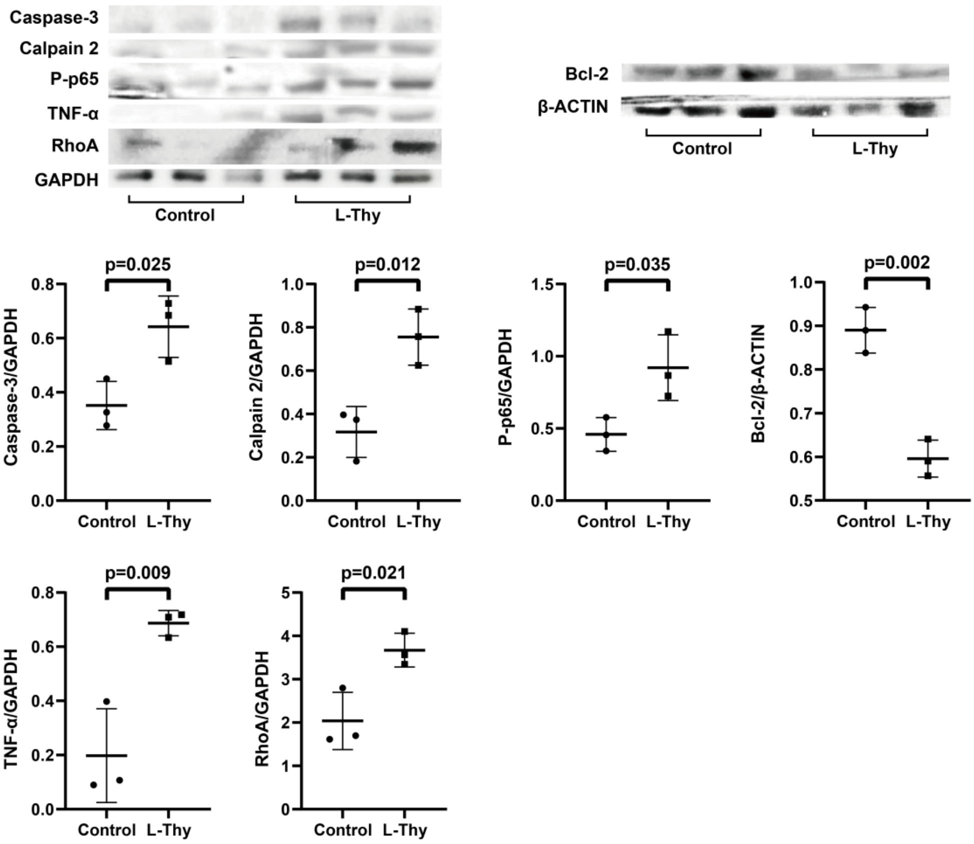

Figure S2. The expression of proteins about signalling pathways in L-Thy induced phenotypes. Caspase-3, Calpain 2, P-p65, TNF-α, RhoA and Bcl-2 protein were detected by WB.

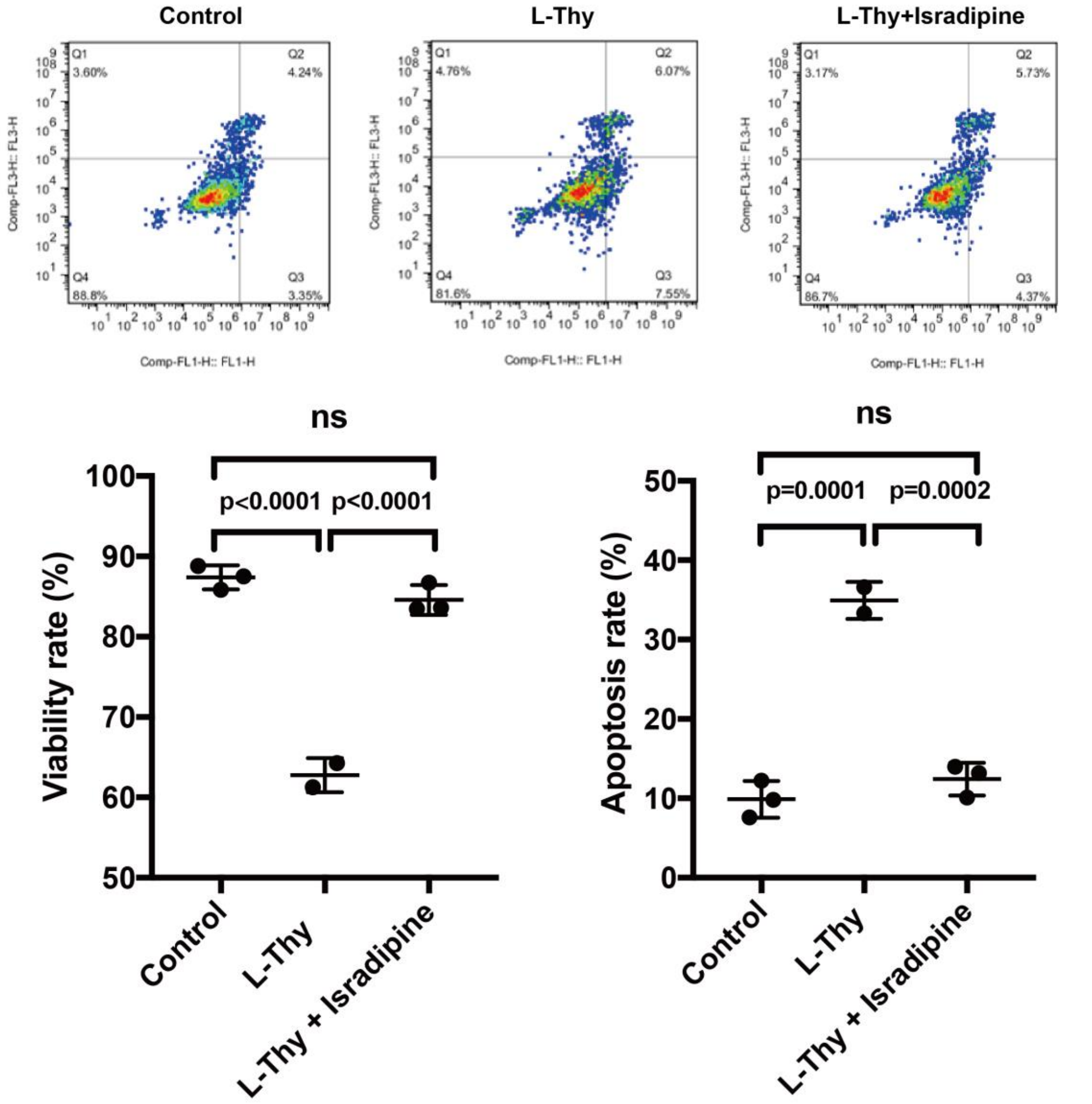

Figure S3. Calcium antagonists (Isradipine) reduce heart muscle damage induced by high thyroid hormones. Typical apoptotic pictures and quantification of the viability rate and apoptotic rate in different groups.
